# “I was so Disconnected”: The Role of Social Bonds in Surviving Childhood Maltreatment

**DOI:** 10.1007/s40653-026-00855-w

**Published:** 2026-03-25

**Authors:** Samantha A. Manuel, Lynne M. Vieraitis

**Affiliations:** 1 Department of Criminal Justice, Fayetteville State University, Fayetteville, NC USA; 2 Program in Criminology & Criminal Justice, University of Texas at Dallas, Richardson, TX USA

**Keywords:** childhood maltreatment, social bonds, turning points, life course, protective factors, resilience

## Abstract

Childhood maltreatment literature predicts that victims of childhood trauma are at greater risk of engaging in antisocial behavior throughout adolescence and adulthood. However, some survivors refrain from antisocial behavior and develop into resilient, productive members of society. Based on interviews with 24 voluntary participants aged 26 to 70 years (*M* = 43.3 years, *SD =* 11.3 years), we explore the turning points throughout the life course to understand how social bonds were broken and rebuilt to prevent antisocial behavior. The findings suggest that protective factors to social bonds established earlier in the life course increased the likelihood that survivors of childhood maltreatment refrained from participation in antisocial behaviors through adulthood. Participants’ attachments to external emotional supports, commitment to their future and personal goals, involvement in prosocial activities, and belief systems were all described to be deeply impactful on inhibiting participation in antisocial behavior. Furthermore, the protective factors of social bonds naturally intersect with one another, and can have an amplifying and compounding influence on each other. These protective factors highlighted the intersectionality of social bonds as they promote resilience in survivors of childhood maltreatment.

## Introduction

According to the United States Center for Disease Control and Prevention (CDC) (2024), childhood maltreatment, defined as emotional, verbal, physical, and sexual abuse and neglect, impacts approximately 1 in 7 children each year in the United States. Because many cases go unreported, this is likely an underestimate (CDC, 2024; Herrenkohl et al., 2022). Prior research suggests that childhood maltreatment has serious impacts on youth development and behavior (Watts, 2017; Young & Widom, 2014). Individuals who have suffered from child maltreatment are more likely to perpetuate the long-term effects of their trauma onto others and suffer from a number of neurocognitive and mental health disorders lasting into adulthood (Edwards et al., 2003; Baldwin et al., 2023; Bürgin et al., 2025; Cui et al., 2020; Dube et al., 2003; Gould et al., 2012; Haapasalo & Aaltonen, 1999; Intravia et al., 2012; Kisely et al., 2020; Negriff, 2020). Research suggests that childhood maltreatment significantly impacts the development of social bonds leading to a greater likelihood of antisocial and offending behavior (Anda et al., 2006; Benedini & Fagan, 2018; Currie & Tekin, 2012; Dube et al., 2003; Evans & Burton, 2013; Fang et al., 2012; Felitti et al., 1998; Intravia et al., 2012; Özdemir Bişkin, 2024; Sousa et al., 2011). Individuals who suffer childhood maltreatment are also more likely to engage in a “cycle of violence”, demonstrated by anger, frustration, and verbal or physical aggression toward their peers, partners, or children (Eriksson & Mazerolle, 2015; Herrenkohl et al., 2022; Özdemir Bişkin, 2024; Widom, 1989, 2017). Yet, despite the increased risk of child victims perpetuating a cycle of violence, some individuals demonstrate resilience, and refrain from serious antisocial behavior (Afifi & MacMillan, 2011; Cerdeña et al., 2021; Collishaw et al., 2007; Cui et al., 2020; Leung et al., 2022; Mc Gee et al., 2020; Meng et al., 2018; Tyler et al., 2008; Ungar, 2013).

The current study aimed to explore the role of social bonds, protective factors, and resilience to criminal behavior among survivors of childhood maltreatment (Hirschi, 1969). To accomplish this goal, data were drawn from virtual face-to-face interviews with 24 voluntary participants aged 26 to 70 years (*M* = 43.3 years, *SD =* 11.3 years) who had experienced childhood maltreatment including physical and sexual abuse and neglect. The data include their experiences as victims of childhood maltreatment, the role of social bonds, and the reasons they refrained from various forms of antisocial behavior. In the next section, we review the theoretical and empirical literature on Hirschi’s (1969) social bonds theory and childhood maltreatment. The data were also analyzed in alignment with Laub and Sampson’s (2003) age-graded theory of informal social control, to highlight the “turning points” in survivors’ social bonds throughout the life course. This is followed by a discussion of our methodology and findings. Lastly, we interpret our findings and offer insight for potential policy implications and future research directions.

## Background

### Childhood Maltreatment and Social Bonds

There are many forms of childhood maltreatment, ranging from physical, emotional, and sexual abuse, and neglect, to witnessing violence perpetrated by one parent against the other (Ben-David & Jonson-Reid, 2017; Greene et al., 2020; Kim et al., 2017; Silvern & Griese, 2012). Often children are victims of multiple types of abuse at the same time, resulting in a compounding effect on child development (Butler et al., 2020; Collishaw et al., 2007; Currie & Tekin, 2012; Daníelsdóttir et al., 2022; Edwards et al., 2003; Kim et al., 2017; Negriff, 2020; van der Put et al., 2015). According to social bonds theory, the risk of deviant and criminal behavior is higher among those who have damaged social bonds, such as those who have experienced childhood maltreatment (Costello & Laub, 2020; Hirschi, 1969; Tyler et al., 2008; Ward et al., 2015). Primarily formed in the early years of childhood, these social bonds include *attachment*—the emotional closeness to others; *commitment*—appropriate lifestyle aspirations and goals; *involvement*—participation in conventional activities; and *belief*—acceptance of the validity of rules and laws (Hirschi, 1969). When these four elements are present, children are more likely to engage in lawful and prosocial behaviors; however, when the bonds are weak or damaged, they are more susceptible to antisocial and criminal behavior.

Evidence suggests experiences with childhood maltreatment in the early stages of the life course interrupts the development of social bonds, particularly attachment, which in turn directly influences the other three elements (Dion et al., 2016; Intravia et al., 2012; Zhang et al., 2026). Attachment bonds are frequently cited as the most impactful social bonds throughout the life course, and can be predictive of future offending behavior and victimization (Ban, & Oh, 2016; Collishaw et al., 2007; Currie & Tekin, 2012; Lowell et al., 2014; Özdemir Bişkin, 2024; Su et al., 2022; Tyler et al., 2008; Widom et al., 2018; Wright & Folger, 2017; Zamir, 2021; Zhang et al., 2026). Furthermore, commitment, involvement, and belief are shaped and impacted by children’s access to resources, which are primarily established by parents and guardians (Collishaw et al., 2007; Wright & Folger, 2017). Thus, the effects of childhood maltreatment on social bonds are exacerbated by stressors such as caregiver employment status, household annual income, high risk environments, and cultural challenges (Baldwin et al., 2023; Bradley et al., 2011; Dubowitz et al., 2016; Monnat & Chandler, 2015).

### Childhood Maltreatment, Antisocial Behavior, and the “Cycle of Violence”

Research on childhood maltreatment suggests such disruptions in the development of social bonds results in a greater likelihood of future offending behavior (Currie & Tekin, 2012; Watts, 2017; Widom, 1989, 2017). Childhood maltreatment experiences may hinder neurological development, which can contribute to the development of mental health disorders such as anxiety, depression, and post-traumatic stress, and substance abuse disorders (Anda et al., 2006; Edwards et al., 2003; Baldwin et al., 2023; Bürgin et al., 2025; Cui et al., 2020; Dube et al., 2003; Felitti et al., 1998; Gould et al., 2012; Greene et al., 2020; Haapasalo & Aaltonen, 1999; Intravia et al., 2012; Kisely et al., 2020; Kramer et al., 2008; Li et al., 2023; Negriff, 2020; Widom et al., 2018). Experiences of childhood maltreatment have been linked to impulsivity, insensitivity, risk-taking, and short-sightedness, resulting in antisocial or criminal behavior during adolescence and early adulthood (Currie & Tekin, 2012; Intravia et al., 2012; Monnat & Chandler, 2015; Özdemir Bişkin, 2024; Tyler et al., 2008; Watts, 2017).

The literature on the “cycle of violence” supports the view that broken bonds resulting from childhood maltreatment, especially those of attachment, make it difficult for victims to form positive interpersonal relationships with others (Ban & Oh, 2016; Butler et al., 2020; Collishaw et al., 2007; Currie & Tekin, 2012; Greene et al., 2020; Herrenkohl et al., 2022; Lowell et al., 2014; Özdemir Bişkin, 2024; Su et al., 2022; Tardif-Williams et al., 2017; Widom, 1989, 2017; Zamir, 2021; Zhang et al., 2026). This impairment of social and relationship functioning in victims increases their risk of revictimization via intimate partner violence during adulthood (Butler et al., 2020; Eriksson & Mazerolle, 2015; Herrenkohl et al., 2022; Tyler et al., 2008; Widom, 1989, 2017; Widom et al., 2018). Individuals who experience childhood abuse and trauma are also significantly more likely to maltreat their children (Collishaw et al., 2007; Greene et al., 2020; Haapasalo & Aaltonen, 1999; Herrenkohl et al., 2022; Islam et al., 2023; Marganski, 2013; Tyler et al., 2008; Widom, 1989; Widom, 2017; Widom et al., 2018). In fact, this transmission of similar experiences between generations has been observed in approximately one-third of childhood maltreatment victims (Haapasalo & Aaltonen, 1999). By experiencing childhood maltreatment as a child, adults may develop the belief that violence is an appropriate response to certain situations (Currie & Tekin, 2012; Greene et al., 2020; Marganski, 2013). This behavior has the potential to perpetuate the victimization of the next generation of children, therefore causing a cycle of violence and childhood maltreatment into the future (Greene et al., 2020; Herrenkohl et al., 2022; Marganski, 2013; Widom, 1989).

### Turning Points and Resilience to Childhood Maltreatment

While it has been observed that people who engage in antisocial behavior have higher rates of childhood maltreatment victimization, not all who are victimized offend (Afifi & MacMillan, 2011). In fact, despite the effects of childhood maltreatment on behavior in adolescence and early adulthood, some individuals refrain from antisocial and criminal behavior. Resilience has been defined as “adaptive functioning and/or absence of psychopathological symptoms”, as well as “the ability to withstand, rebound, maintain stable equilibrium, or positively adapt from disruptive life challenges” (Meng et al., 2018; Watters et al., 2023). Protective factors to resilience have been identified across the literature to help survivors of childhood maltreatment respond to life adversities and stressors (Afifi & MacMillan, 2011; Cerdeña et al., 2021; Cho et al., 2024; Leung et al., 2022; Li et al., 2023; Mc Gee et al., 2020; Meng et al., 2018; Wright & Folger, 2017). Protective factors can be found at the individual, familial, and community levels. Examples of such protective factors primarily include prosocial personality traits, spirituality, self-efficacy, self-identity, positive coping skills, family coherence and support, religious communities, school programs, and neighborhood cohesion and safety, and access to resources such as basic necessities, healthcare, and financial opportunities (Afifi & MacMillan, 2011; Ban & Oh, 2016; Cerdeña et al., 2021; Cho et al., 2024; Li et al., 2023; Mc Gee et al., 2020; Meng et al., 2018; Tyler et al., 2008; Ungar, 2013; Waldron et al., 2018; Wright & Folger, 2017; Zhang et al., 2026).

The described protective factors of resilience have inherent alignments with one’s social bonds, as defined by Hirschi (1969). Thus, the increased presence of such protective factors may reduce the impacts of childhood maltreatment experiences on social bonds, and instead promote the development of resilient tendencies. Resilience can be very influential on one’s ability to refrain from potential antisocial behavior related to childhood maltreatment. Even with experiences of childhood maltreatment, survivors may still overcome adversities later in life, indicating the effects of childhood maltreatment can be pivoted, even reversed over time (Laub & Sampson, 2003; Mc Gee et al., 2020; Ungar, 2013). The influences of protective factors and resilience on social bonds over the life course are aligned with Laub and Sampson’s (2003) age-graded theory of informal social control. The negative consequences following childhood maltreatment can be most effectively prevented or mediated if such protective factors are provided early in the life course (Cerdeña et al., 2021; Laub & Sampson, 2003; Mc Gee et al., 2020; Meng et al., 2018). This is known as a “turning point”, which may result in the redirection or reconstruction of survivors’ social bonds (Laub & Sampson, 2003; Wright & Folger, 2017). This in turn increases survivors’ resilience and likelihood of refraining from engaging in antisocial behaviors and the cycle of violence in the future (Afifi & MacMillan, 2011; Cerdeña et al., 2021; Meng et al., 2018; Ungar, 2013). Thus, the authors aimed to explore how childhood maltreatment survivors’ bonds are rebuilt, pivoted, and evolved over the life course through the lens of Hirschi’s (1969) social bonds theory in combination with Laub and Sampson’s (2003) age-graded theory of informal social control.

## Methodology

### Characteristics of Study Participants

The 24 voluntary participants of this study ranged in age from 26 to 70 years old (*M* = 43.3 years, *SD =* 11.3 years). The majority, 71% of participants, identified as White, 25% as Latino/a, and 4% (one) participant, as Black. 71% were female, 25% were male, and 4% (one) identified as non-binary/trans. Sixteen participants (67%) had never had any contact with the criminal justice system, whereas eight (33%) had been arrested and/or convicted of minor crimes ranging from disorderly conduct to petty theft as juveniles. Twenty-three participants (96%) stated that they had not participated in any non-traffic crimes. One participant (4%) admitted to heavy illicit drug use within the past two years but had no contact with the criminal justice system.

### Measures and Procedure

Data were collected from participants at a local therapy and support group specializing in childhood maltreatment and trauma in a large metropolitan area. Approval for the study was obtained from the University’s Institutional Review Board before the recruiting process.^1^ Information advertising the study was distributed by the support group and potential participants volunteered to contact the researchers to indicate their interest in participating in the study. Prior to participation, individuals were emailed a consent form via RedCap explaining the purpose of the study and eligibility criteria. To be eligible to participate, persons met two criteria: (1) experienced past child maltreatment and trauma—physical, emotional, verbal, or sexual abuse or neglect during childhood, and (2) were not engaged in criminal behavior currently as adults. If eligible, participants were sent a unique RedCap survey link to complete the consent process online.

The first author conducted virtual face-to-face interviews with the 24 voluntary participants, all of whom had extensive histories of childhood maltreatment including sexual, physical, verbal, and psychological abuse and neglect. Each interview took place online through a virtual platform due to restrictions placed on researchers during the COVID-19 pandemic and lasted approximately an hour to an hour and a half. The interviewer followed an interview guide (see Appendix), but questions were open-ended to allow participants to provide details and information that may not be captured in a structured interview. Participants were asked questions about past childhood maltreatment, including their relationship to the offender and their perceptions of the impact of their victimization on their childhood, adolescence, and adulthood.

Questions were constructed to explore how their victimization affected their social bonds, as defined by Hirschi (1969). For example, participants were asked to describe their attachments to others, their involvement in extracurricular activities, their commitment to future goals, their belief systems, and participation in antisocial behavior throughout the life course (e.g., “Describe how you believe that your past child abuse and/or neglect affected your quality of life during adolescence/adulthood so far. Can you give specific examples and details such as your job, home, or relationships?”). Participants were also asked questions to investigate the impact of social bonds on their resilience, or their potential continuation of the cycle of violence (e.g., “Have you ever abused or victimized someone? Has your abuse ever caused you to abuse or violate other people?”) (Widom, 1989, 2017; Widom et al., 2018). The interviews were concluded with demographic questions related to age, gender, race and ethnicity, marital status, religion, and education. The interviews were audio recorded, and were manually transcribed by the first author. The transcriptions were then reviewed for quality and accuracy by the second author. All names and other identifying information divulged by the participants were replaced with pseudonyms to ensure anonymity. Once transcription was reviewed by the authors, the audio recordings were destroyed for participants’ confidentiality.

### Past Childhood Abuse and Neglect

Participants reported a wide range of childhood maltreatment including emotional, physical, and sexual abuse. Nineteen individuals had experienced sexual abuse, usually in combination with some other form of abuse, such as verbal abuse or emotional manipulation. Consistent with prior research (Hurren et al., 2018; Santhosh, 2016), offenders were typically people who assumed guardianship or mentoring positions in the victims’ lives and included primary family members such as fathers, stepfathers, and brothers, extended family including aunts and uncles as well as babysitters and neighbors. Most participants reported a single perpetrator throughout their childhood and adolescence but a few experienced victimization from multiple offenders.

In addition to sexual abuse, many participants reported high levels of emotional neglect—feelings of being unwanted, unloved, and isolated. This form of maltreatment was commonly associated with verbal abuse. Participants experienced verbal abuse both directly, i.e., a parent would yell, berate, and belittle the child; or indirectly, as the child witnessed the verbal abuse of one parent by the other. Physical abuse was also direct—a child was physically assaulted by a parent—or indirect—the child witnessed the physical assault of a parent or sibling; in some cases, victimization was both direct and indirect. The incidents of child maltreatment experienced by participants in our study highlighted the abusive, stressful, and unsafe environments in which they lived during childhood and adolescence. The extent to which such negative life events impacted their relationships with parents and others is the subject we examine next.

### Coding Method

The interview data were read line by line and coded by hand, identifying the four elements of the social bond as identified by Hirschi’s (1969) social bonds theory. This process was performed by the researchers independently for each interview transcript. Upon completion, the authors met to discuss the coding and resolve any disagreements. The goal of this research was to understand how bonds of attachment, commitment, involvement, and belief were related to child maltreatment and behaviors in childhood and adolescence. Participants explained how their victimization affected their relationships with others (attachment), their participation in extracurricular activities (involvement), their work ethic in relation to education or career (commitment), religiosity and spirituality (belief) and participation in antisocial behavior from childhood to adolescence.

During the coding process, the authors noticed parallels to Laub and Sampson’s (2003) age-graded theory of informal social control, which emphasizes the importance of bonds throughout the life course and not just during childhood and adolescence. Although their research focuses on explaining criminal careers, this theory was used as a framework to understand how participants’ social bonds changed over the life course, and we use the concept of turning points to highlight these changes (Hirschi, 1969; Laub & Sampson, 2003). Thus, the findings are organized to show how child maltreatment impacted the social bonds of attachment, commitment, involvement, and belief and how turning points led to the establishment or repair of bonds and the resistance to antisocial behavior.

## Findings

### Attachment

Consistent with prior research, the childhood maltreatment experienced by participants had negatively impacted relationships with others throughout their life course (Anda et al., 2006; Demers et al., 2019; Dube et al., 2003; Felitti et al., 1998; Herrenkohl et al., 2012; Lowell et al., 2014; Neil et al., 2022; Özdemir Bişkin, 2024; Su et al., 2022; Tardif-Williams et al., 2017; Widom et al., 2018; Zamir, 2021). The physical and emotional trauma of abuse had its most immediate impact on participants’ relationships with parents and guardians, however, their ability to form bonds with others suffered as well (Lowell et al., 2014; Özdemir Bişkin, 2024). Sandra, who was sexually abused by a family member entrusted by her mother, said, “I just feel like she broke the bonds between us, you know, by leaving, and I feel like I probably steeled my heart and try to protect myself from getting hurt again.” Sandra’s reaction was typical. Participants described having distant or surface-level relationships with friends, classmates, and peers; issues with trust were common.

**Trust issues and fear.** Most of our participants described feelings of social isolation and distrust. The participants noted they could not open up to their friends or peers, because they feared rejection or a lack of empathy, should the person become aware of the abuse (Lowell et al., 2014; Widom et al., 2018; Su et al., 2022; Zamir, 2021). As Rene explained, the sexual, physical, and emotional abuse inflicted by her mother and father from age five to 15, affected her ability to connect with others:

I think that [the abuse] isolated me from people, …I became very skilled at hiding things… When people would question me, …I would give them what they wanted to hear versus the truth, because the truth was too painful to say. …It was hard for me to make friends, you know, I didn’t have the skillset to make those friends when I was growing up, because I was always so afraid they were going to find out something.

Lily’s account was similar as she recounted the trust issues and fear associated with having friends, “I never wanted to tell anybody anything, ‘cause I was just sort of so afraid of, of people sharing anything that I felt like was a secret. So, just really deep trust issues.” Others claimed to have a lot of friends, but, again, a lack of trust characterized most of these relationships. As Selena, who was sexually abused by her stepfather, explained:

I think that [the childhood abuse] really screwed me up in the sense of trusting anybody or letting them in from the outside. I was a really good faker, like a good actress, in the sense that I was nice to everybody, and kind to everybody, and always had a ton of friends, and, but- I was also like, I always wanted to protect [myself].

Sandra also talked about having friends but exhibited caution in her interactions, as she explained, “I would not open up, you know? I wouldn’t talk about my feelings, I would always ask questions, and talk about other people, but not open up about myself.”

Fear of discovery and issues of trust were common among participants as they described their relationships during childhood and adolescence, but for many, problems with attachment to others persisted through adulthood (Lowell et al., 2014; Su et al., 2022; Widom et al., 2018; Zamir, 2021). For some maintaining adult relationships was difficult as they kept partners at a distance and rarely trusted that a partner would stay. Sandra’s account was common, “…I didn’t have a problem meeting people… I kept people at a safe distance and never let them in… If I felt like they were going to leave me, I left first, ‘cause it’s like I always wanted the upper hand.” Matt described similar experiences with relationships, “I can have very intimate relationships with people on an emotional level. I cannot have, I have really bad sexual intimacy issues, I’ve never been in a long-term relationship.” Riley described the impact of the abuse on their marriage: “…I have a hard time connecting with and expressing pain, needs, letting other people help me, or letting other people. …And sometimes, I struggle with touch aversion.” Similarly, Jorge explained the difficulty of building relationships with others:

I did [have a] really hard time building relationships and opening up to people. To this day, I have a very hard time telling people what I feel or need. I’ve become really good about guessing what they want to hear or want me to do.

**Building attachment.** Despite having deeply rooted trust issues, some participants talked about individuals with whom they formed attachments and could confide in about their abuse. These social ties often came about through respondents’ involvement in other prosocial behaviors during childhood and adolescence, such as extracurricular activities, hobbies, and school, and later in life through employment, intimate partner relationships, and support groups. These attachments mediated the effects of childhood trauma and helped nurture participants’ ability to trust and connect with others (Lowell et al., 2014; Ungar, 2013; Wright & Folger, 2017; Zamir, 2021). These attachments had significant impacts on participants’ lives and many described them as “turning points” that helped them build resilience and to develop and strengthen other social bonds (Ban & Oh, 2016; Laub & Sampson, 2003; Su et al., 2022; Thornberry et al., 2013; Zamir, 2021; Zhang et al., 2026).

Because of the weakened attachment to immediate family members, some participants relied more heavily on other relationships, such as teachers, extended family, or friends; and later in life, on relationships with significant others, coworkers, and support group members (Ban & Oh, 2016; Intravia et al., 2012; Su et al., 2022). Twelve participants identified teachers as playing a significant role in their lives. For example, Leslie said, “I had a few, really kind teachers, who I latched onto every single one of them, at the time they were in my life…like, a mother figure, like, an actual, kind, nurturing, mother figure.” Tiffany also talked about the influential role teachers had in her life:

… Anyone who would extend their hand, I took it, and, and I’ve been, ‘til around from 6th or 7th grade, I adored my teachers. I just would latch on to anyone who would teach me, or kind of see me as a person that wasn’t defined by what was happening at home.

Others, like Allison, found support from friends’ parents, “So, I’ve had a lot of friends through the years, that their moms became like a mom to me, and I think, it’s because they all knew, kind of, what I grew up with.” Leslie explained the importance of her relationship with her grandmother as well as her aunt:

I was very, very close with her [grandmother]. …not close enough to tell her about any of this, but I adored her, and I cherished every moment that we were together. …I also viewed her as—kind of viewed my grandmother like a mom, and another aunt that I had, I kind of viewed her like a second mom.

Despite their histories of childhood abuse and neglect, participants who had adults in their lives with whom they formed healthy attachments, were able to avoid engaging in serious forms of antisocial behavior. In addition, these relationships helped facilitate the development of attachment to others in adulthood through employment and other activities, as well as through relationships with intimate partners. Many of them described an individual or a few people who helped them overcome their issues with trust and provided a nurturing environment that facilitated their attachment to others. For example, Allison, whose mother was addicted to drugs and neglected her, found stability in her partner and her partner’s family. She explained that her partner is, “a wonderful person, her mom is a wonderful person, and I feel like I’m part of their family. I feel like her mom is like a mom to me, and she does the things that a mom is supposed to do.”

Many individuals discussed how their fellow employees who are also their friends helped mediate the long-term effects of childhood abuse and trauma in their adulthood. For example, Matt stated that he has confided in these people everything about his past abuse and can always rely on them for support. He explained how important his friends are to his quality of life:

I have three guys who I consider my family of choice. To use layman’s terms, they are my three best friends. They know everything about me… Each one of those friendships brings something different to my life… I think I am blessed with friendships. I think all of those things, taken together, are a fortress, to keep me safe, and from making, not making too many bad decisions.

Cameron also described a mentor in her place of employment who helped guide her during a difficult time and keep her “straight”:

I ended up starting to work at a salon where… I ended up becoming best friends with the owner of the salon. …she was so empowering to me, like, ‘you can do this, I can help you do this’… I think she was very, very influential in helping me, in helping guiding me in the right direction. Because at the time, you know, I was rebellious against my mother, but she was more of a guiding hand, if that makes sense… She helped guide me in, you know, keeping me straight.

Participants’ accounts revealed the extent to which the bond of attachment, which should have been established and fortified by parents and guardians, was impacted by child maltreatment. As reported by participants, common outcomes of the lack of attachment included significant distrust of others, fear of relationships, and social isolation. However, participants also described how the bond of attachment was built and nurtured by social supports, i.e., people with whom they interacted such as teachers, outside of their homes regularly over the life course (Ban & Oh, 2016; Thornberry et al., 2013; Su et al., 2022; Wright & Folger, 2017). Participants pivoted to seek attachment bonds from others to supplement the lack of relationship with their parents and guardians (Laub & Sampson, 2003; Wright & Folger, 2017). It was these attachments that participants cited as helping them build trust in others and the desire for connections and facilitating involvement in prosocial activities, rather than deviant and criminal behavior.

### Involvement

One of the most common ways participants were able to avoid antisocial behavior, as well as deal with their victimization, was through involvement in extracurricular activities. In childhood and adolescence, participation in fine arts, sports, and school clubs was often recalled as providing a safe space and time away from abusers, as well as outlets for emotional release (Cho et al., 2024; Su et al., 2022; Tyler et al., 2008). Moreover, since many of these activities were tied to their schools, participants were presented with additional opportunities to form friendships and connections with others (Ban & Oh, 2016). Participation in activities also helped foster their commitment to their futures through engagement in their schools and communities.

**Minimizing time at home.** The most common reason for involvement in extracurricular activities was to avoid being at home for as much time as possible. Sofia, who participated in many teams and clubs, explained:

Once I got to the point where I could, …I would just, like, try to be not home as much as possible… Since the 4th grade through high school, like, every single year, I was in some type of choir or theater, so, I would always have, like, rehearsals or practice. …I was in soccer for a little bit in high school… And then I would get myself involved in… after school programs or something.

Leslie voiced similar reasons for her involvement in music, sports, and dance even if she didn’t always love participating in them:

I did a lot of things to try to not be at home. Like it’s a combination of, I’m just kind of curious… and anything I could do to have an excuse to not be at home, was something I would take. …I definitely enjoyed those things, too, …And some of those other things, I liked them, but I didn’t love them… I definitely tried to keep busy.

**Emotional outlets.** Participants also described their involvement in activities as an emotional outlet for their anger and frustration over the abuse (Cho et al., 2024; Tyler et al., 2008). Several participants, like Tonya, cited singing and music programs as important outlets, as she explained, “music grabbed me… it was the rhythm that helped me…get…some of my frustration out.” Allison also described the role music played,

I kind of would come home and play my guitar every day. …My older sister played guitar, she kind of taught me the chords. And we sang, and then I ended up singing at church, and singing in weddings. …The music was kind of my outlet. I listened to a lot of music all the time.

For others, involvement in sports teams allowed them to direct their anger at an appropriate target. For example, Lindsey described softball as “a pretty good outlet, just basically, just hitting the ball with the bat, was pretty- it helped to relieve a lot of anger.” Riley’s account was similar, as they were involved in sports to “get some feelings out.” Jade cited cheerleading as her “escape” and a way to release anger, “…tumbling isn’t like boxing, but you know. Like, it’s just kind of like a high-impact sport. Like, tumbling kind of, like, got your anger out.”

Sports teams were also essential to building social relationships with peers and adults as participants often spent time at their teammates’ homes after practice. Danny, an avid soccer player, explained the importance of soccer for his mental health as well as for his relationships with others:

I think I had an identity, really, from an early age. I was an athlete, and I had, I used it really well. …It was…good for my self-esteem, helped me feel like a part of something, made me feel like I was valuable. I built a lot of good relationships, friendships I had growing up were through soccer, so that was, that was really important to me.

As previously mentioned, the many activities that individuals participated in during their youth and adolescence were often directly related to their engagement in school and their commitment to education. They offered important channels for minimizing contact with their abusers, providing emotional outlets, and facilitating relationships, as well as avoiding antisocial behavior.

**Continuing involvement.** As participants transitioned to adulthood, many maintained their involvement in activities, as they continued to provide an outlet for or diversion from the emotional trauma of their childhood experiences, as well as help them connect to others. For example, Allison described how her passion for music has always been a way for her to connect with others, as well as a way to express her feelings and promote healing:

I continued to play my guitar, and sing with friends or my sister. And I would sing if we had, like, a get-together, or party, or something at the house. Music, as far as just listening to music, I feel like it’s been an outlet for me always. And I try to write some songs, and just put my feelings down on paper. So, yeah, it’s been a pretty big part of my life.

Danny, who played soccer from childhood through college, also talked about how his involvement with the sport continued to be an important outlet for him as well as a way to maintain and foster relationships, “I do watch a ton of soccer, …I got guys here, we watch matches together.” Logan explained how his hobbies continued to prevent him from engaging in antisocial and deviant behavior:

I read comic books, and I drew, I painted, that eventually led to woodworking, and furniture making, you know? Basically, like, I, I am really good with my hands, for lack of a better way to say it. … If I’m, if I’m not engaging in this behavior, then I’m going to be doing something bad, you know what I’m saying?

Respondents also maintained their involvement in educational and employment pursuits as higher education and careers became important as they aged. They were very involved in the success and productivity of their careers as a source of fulfillment and satisfaction. For example, Joyce, who was a good student and involved in many school extracurriculars, explained how this commitment carried over into adulthood and led to her involvement in philanthropic work:

I made all straight As in everything except algebra. …I was in choir, I was in Brownies, I was an officer in student council, I was a cheerleader…active in youth groups…had a lot of leadership [positions]. …I have been board chair of [a local charity foundation], which raises money for women and girls… I co-chaired a campaign [that raised] …the most that any woman’s group in the country had ever raised.

Jack also elaborated on how important his involvement with his community was to his personal healing journey:

And as soon as I can remember, I was volunteering or coming up with some sort of fundraiser for something or someone, you know? Any sort of injustice. I’ve sat on several boards and committees and whatnot throughout my entire life. …Being a part of something like those organizations, various organizations all across the board, or just creating events, you know? It just kind of showcases, you know, where the heart is, your true, true character….

In sum, participants described the importance of opportunities to involve themselves in prosocial activities and extracurriculars. By minimizing the time spent at home, respondents were able to reduce the likelihood of victimization and find suitable outlets for their emotions. These diversions also served as emotional releases that promoted mental health, self-esteem, and self-expression. Engagement in their schools and communities during childhood and adolescence facilitated continued participation in such positive behaviors into adulthood, subsequently reaffirming their commitment to their futures (Cui et al., 2020).

### Commitment

Prior research has shown that some victims of childhood maltreatment may turn to antisocial behavior as an outlet or coping mechanism (Benedini & Fagan, 2018; Cui et al., 2020; Özdemir Bişkin, 2024; Sousa et al., 2011). Other research suggests that early engagement with antisocial activities can be an indicator of a lack of commitment to one’s future livelihood and success (Afifi & McMillan, 2011). Participants in our sample, although they did not engage in antisocial activity as adolescents, did commit some minor offenses. For example, Taylor and Joyce both mentioned stealing change from their parents or “swiping candy” from convenience stores. The majority of the participants, however, did admit to drug use during their adolescence and early adulthood but much of it occurred in social settings, such as with friends or at parties.

**Work ethic in school and career.** While many participants refrained from criminal behavior out of fear of more abuse, “I’m afraid to do anything that is going to get me into any trouble… I just, I don’t want to be in trouble” (Lindsey), most cited their commitment to doing well in school and later in adulthood at work. Rene’s account reflected both the fear of getting in trouble and her commitment to doing well in school:

I was well behaved, I didn’t make trouble, I wasn’t a troublemaker. …I was a very good student, I made good grades, I was like, on the honor roll, stuff like that. I guess I had enough trouble at home, I didn’t want trouble at school… [The abuse] made me extremely strong, determined, tenacious, …it made me very driven, driven to succeed.

The drive to succeed was also identified in Tiffany’s account on the importance of doing well in school, “I was an avid reader… I can’t remember what the book club was called… the one that read the most books, and would win the grade pizza party, and then my whole family would get Six Flags tickets.” In addition to a strong commitment to education, many participants emphasized the importance of their work and careers for providing sources of self-worth and fulfillment in adulthood (Cui et al., 2020). On how her abuse as a child impacted her work ethic, Lindsey said:

I have a pretty strong worth work ethic. I don’t know exactly how the abuse has, like, affected that, as far as- the only thing I can see is, like, because, I don’t like to make mistakes, I work really, really hard to ensure that I don’t. …I think because I feel like I have to prove something, that I work extra hard.

Leslie’s description of her career was similar, but she also admitted that the drive to work hard could be problematic as it was a way of avoiding memories of childhood trauma:

I’m a hard worker, I’m very hard on myself, I’m a perfectionist… that’s something more recently, that I’m trying to back off, …trying not to work a million hours, and trying not to prove my worth via my productivity. …[Backing off] is hard, because, that idea of keeping busy at a company, out of some of my memories, for a time, and I think I just didn’t realize, I needed to shift coping skills.

Tiffany also cited past maltreatment as the impetus for a strong commitment to work hard. Although it was a positive characteristic, she also acknowledged there were negative aspects:

In terms of work ethic, though, I think there is a constant fear of disappointment. …I’m always referred to as “the workhorse”, and I tend to- and, and this is both maybe positive and maybe downfall, but, I tend to be the person that is given these lofty, major projects, that, you know, everyone is putting all their bets on, and it also looks insurmountable, like, it’s just on fire. And for better or worse, the reputation is, “well, [Tiffany] knows how to figure things out”, and so, I do think a lot of the kind of resilience that emerged from childhood, created that, almost kind of scrappy, “you can make anything you want out of nothing” mentality.

The need to be independent and succeed was common among participants. As Lily said: “I just had a really strong drive to, to be successful, and independent, and kind of be able to stand on my own, without anybody else being able to have input or influence into my life.”

**Building commitment.** For participants, who were engaged in minor antisocial behaviors in their youth and adolescence, the opportunities to develop strong work ethics through school and employment were transformative for building their commitment to their future outcomes. While often motivated by a desire to partake in activities and behaviors that inhibited the traumatic feelings associated with abuse and neglect, the turning points associated with the bonds of commitment primarily arose when they were emerging into young adulthood (Cui et al., 2020; Cho et al., 2024; Laub & Sampson, 2003; Li et al., 2023). It was at this stage of their lives that many were finally able to eliminate their interactions and associations with the sources of their trauma. For some, like Tiffany, the turning point came in response to a serious life event. She described why she felt that she had a second chance to do better for herself:

I attempted suicide, and the police showed up and took me to a mental hospital, and I waited there for about, twelve hours for intake. …I changed high schools, and it was almost like a second life. …I’ve always trusted that I need to stay on the straight and narrow, ‘cause, I was given a second chance to live.

For participants who did not engage in any antisocial behavior during their adolescence, turning points occurred much earlier in their lives. Exposure to the traumatic behavior displayed by parents and other adults led many participants to become extremely averse to engaging in such behaviors themselves. Their experiences with maltreatment and witnessing antisocial behavior reaffirmed their commitment to their futures and solidified their desire to be successful and not continue a cycle of abuse and neglect.

### Belief

All the participants in the sample were raised in affiliation with a religious institution. This is due in part because they were drawn from a population in a Southern state in the “Bible Belt” where it is common for families to use religion to instill moral values and principles in their children (Brunn et al., 2011; Waldron et al., 2018). However, participants’ receptiveness to and acceptance of their religious upbringings varied widely.

**Religion and spirituality.** Some participants were deeply connected with organized religion or acknowledged a spiritual being or God. Whereas, for others, their experience with religion was associated with their abusers and physical and emotional trauma (Waldron et al., 2018). For example, Dominic described how religion was a positive force and guide throughout his life course:

Not everything that I’ve been through would have been possible without God. The only way I’ve been able to continue not by my own will but just continue and go forward, yes, because of God. And when I say God, I mean a benevolent, loving, caring, empathetic, sympathetic, understanding, all-knowing, powerful force of love. I wouldn’t be here today. I would be dead or in the prison if it had not been for that.

Lacy reflected a similar belief about the importance of faith as a child when she talked about her views on religion, “my mom would always say ‘this is our cross we’re carrying, and God is here to help us, and we won’t fail with Him’. I really valued my faith, when I was growing up, ‘cause it really helped me.” Esmerelda’s account was similar, in that she “…always had a strong faith, always, even when things were really, really horrible.” As she explained, “ it has got me through every situation in my life. …I have always had a very strong, and I would say more spirituality and relationship with God directly.”

In contrast, other participants rejected their religious upbringing, citing its connection to their victimization. As Sofia explained the role of the church in her experience:

I was never very religious. I just did it [go to classes] because I was forced to go, but I was- I think, you know, my experiences did impact my ability to connect, you know, spiritually and religion, because I was just, like, well, if that, if there was really, like, a God or something like that, like, I mean, why would, why would they, why would he put me in a family that would, like, just hurt me in this way, right? So, it shattered my ability to believe, because of just the terrible things that I went through.

**Changes in belief.** As the participants came into adulthood, they described developing their own beliefs about religion and morality. Some participants continued to accept or adjust their beliefs in the religious teachings of their youth, while others rejected them. Allison described herself as being “a little resentful to religion for a while. And then as I got older, I became more, I wouldn’t say religious, but more closer with God.” Her belief became stronger over time, as she explained how God “…took care of me, and He got me through a lot of things in my life. …I know He got me through a lot of things, and helped me through a lot of things, and continues to bless me.” Selena expressed a similar feeling about her belief in God and how her views changed over time:

I am [angry with God], I am because I don’t understand what I did wrong”. Like, I know they tell you that God doesn’t give you more than you can handle, but I’m not a buffet, like, what the heck? And so, it’s part of the reason why I no longer go to the Catholic Church. I think that, because for me, again, we were going to church every Sunday to Catholic Church, and he still did this to me. …And then, when I moved here, I started attending a non-denominational church, and opened the Bible a lot more than I ever had in my whole life. …And so, it’s just given me comfort in knowing that there’s a higher power out there for me.

While some participants maintained their relationship with organized religion, others like Tonya described moving away from the church toward spirituality (Waldron et al., 2018). She explained her beliefs:

There’s a term called “church hurt” …when the religious side of it abuses you. I felt abused at church, as well, by the pastor… [God is] everywhere, He’s not just in a building, He is everywhere. And there’s somebody who doesn’t go in a church that needs to know that there’s hope for life. …Whatever culture you are, whatever name He is to you, He is to you, and He meets you at your level of need.

On the other side of the spectrum, some participants had a very difficult relationship with religion. Sandra’s brother-in-law, who was a pastor, had sexually abused her growing up. Coming from a Christian household, Sandra explained that religion was something she could turn to when she was younger, and she felt “it was really drilled into me, that God loves me, I’m a lovable person, and Jesus loves me, God has plan for your life, and …I do feel like it, kind of propped me up, during those years.” However, as she grew older, her perspective on religion shifted, and she became agnostic. She said:

…It didn’t serve me any purpose, didn’t serve any purpose for me anymore, and, also, some of the beliefs were just pretty extreme. I just couldn’t believe in faith, healing, and certain things, like, why would God only heal- like, why are people sick, then, or I don’t know. Just a lot of things didn’t make sense anymore.

Although all of the participants in this study had experiences with a religious upbringing, their relationships with religiosity through the life course were also shaped by their personal definitions of honor and virtue. Whether the participants found solace in organized religion or spirituality or relied on their personal belief systems of morals and ethics, these bonds did play a role in their resistance to criminal behavior (Waldron et al., 2018). Citing their beliefs as well as their commitment to future success in education and careers, participants intentionally refrained from engaging in serious criminal behavior throughout their life course.

## Discussion and Conclusion

The findings demonstrate the impact of child abuse and neglect on the formation of the social bonds of attachment, commitment, involvement, and belief. As reflected in their narratives, participants’ bonds of attachment were severely damaged by their abusers, leading to serious trust issues and difficulties in forming relationships (Lowell et al., 2014; Mielke et al., 2020; Thornberry et al., 2013; Wright & Folger, 2017). Yet, despite these issues, some participants were able to form relationships with others with whom they could confide about their abuse and related emotions (Ban & Oh, 2016; Lowell et al., 2014; Özdemir Bişkin, 2024; Wright & Folger, 2017; Zamir, 2021). Many of these relationships were facilitated by participants’ involvement in activities as well as commitment to doing well in school and later in work (Cui et al., 2020). These attachments therefore mediated the effects of trauma perpetrated by their abusers, and helped nurture survivors’ trust in and connection to others (Lowell et al., 2014; Meng et al., 2018; Ungar, 2013; Wright & Folger, 2017; Zamir, 2021). As a result, they were able to develop and strengthen other elements of the social bond, an important factor for inhibiting participation in antisocial behavior.

As the data show, most participants were highly involved in their schools, communities, and related activities (Ban & Oh, 2016; Cho et al., 2024; Tyler et al., 2008). Their involvement in extracurricular activities afforded them opportunities to spend time away from their abusers, as well as to cultivate their skills and form attachments to others (Thornberry et al., 2013; Wright & Folger, 2017). Participants’ commitment to improving the quality of their lives went hand-in-hand with their beliefs (Cui et al., 2020; Li et al., 2023). By deciding not to continue the cycle of violence and not to engage in other criminal behavior, participants demonstrated their belief in moral and ethical behavior, while also promoting their future and personal goals to become productive and successful members of their communities (Collishaw et al., 2007; Cui et al., 2020; Heller et al., 1999; Li et al., 2023). By focusing on the other elements of the social bond, they were able to refrain from acting out through deviant or criminal behavior.

In sum, participants’ commitment to their future and personal goals, their involvement in extracurricular activities, and their belief systems were all described as having profound influences on inhibiting their participation in antisocial behavior. For this reason, social bonds acted as protective factors preventing further abuse and trauma during participants’ youth and into adulthood (Afifi et al., 2016; Ban & Oh, 2016; Collishaw et al., 2007; Cui et al., 2020; Li et al., 2023; Waldron et al., 2018; Wright & Folger, 2017; Zamir, 2021). The strength of their bonds of commitment, involvement, and belief mitigated some of the negative effects of their childhood maltreatment and allowed them to grow and develop into successful adults. Their involvement in these outlets therefore resulted in increased attachment bonds to others, who ultimately become a part of a stronger network for these survivors to depend on for emotional support, empathy, and reassurance (Ban & Oh, 2016; Lowell et al., 2014; Wright & Folger, 2017). In other words, the protective factors of social bonds interact with each other and can have an augmenting and compounding influence on one another (Mc Gee et al., 2020; Meng et al., 2018). Many protective factors discussed above highlighted the intersectionality of social bonds as they promote resilience in child maltreatment survivors.

### Building Bonds and Turning Points

During the period between adolescence and young adulthood, the separation from the sources of victimization allows emerging adults to develop their sense of identity and form new social supports outside of the source of childhood trauma (Ban & Oh, 2016; Cui et al., 2020; Latham et al., 2023; Leung et al., 2022; Thornberry et al., 2013; Wright & Folger, 2017; Zamir, 2021). During this fundamental time of one’s identity development, respondents became more aware of how the effects of their childhood maltreatment experiences and associated trauma permeated into other aspects of their lives (Cui et al., 2020; Latham et al., 2023; Taussig et al., 2023). The social bonds developed through early childhood experiences facilitated the opportunity for potential turning points during respondents’ late adolescence and early adulthood (Laub & Sampson, 2003). This is an integral point of survivors’ emergence into adulthood, where they have the opportunity to develop their identity without the prevalent influences (and potential repeat exposure) of their childhood experiences (Leung et al., 2022; Thornberry et al., 2013; Wright & Folger, 2017).

During this period of time, survivors have the opportunity to establish, develop, strengthen, and/or adapt their social bonds with diminished or no interference from the source of their trauma. These turning points provided opportunities for survivors of childhood trauma to find meaning, insight, and self-understanding of their lives (Cui et al., 2020; Laub & Sampson, 2003; Mc Gee et al., 2020; Taussig et al., 2023). For instance, childhood attachments to social supports that encouraged other prosocial behaviors were social ties that participants intentionally continued to cultivate and nourish (Ban & Oh, 2016; Lowell et al., 2014; Thornberry et al., 2013; Wright & Folger, 2017; Zamir, 2021). As these connections developed and grew over time, they introduced the respondents to other sources of social support, providing an opportunity to find meaningful connections to others who enrich and fulfill them (Leung et al., 2022; Lowell et al., 2014; Snijders et al., 2018). Similarly, involvement in extracurriculars that brought diversion and joy during childhood and adolescence helped to simultaneously develop soft skills, give meaning and purpose, and widen their interests in other related prosocial activities going into adulthood. Such turning points provided the opportunity for emerging adult survivors to consciously choose to recommit to their future happiness, and develop a self-identity separate from the trauma experienced during childhood (Cui et al., 2020; Laub & Sampson, 2003; Taussig et al., 2023; Wright & Folger, 2017). All of these experiences help promote hopefulness and optimism, which can in turn help facilitate further resilience and healing (Cui et al., 2020; Taussig et al., 2023).

### Limitations

There are several limitations in the current study which must be considered when interpreting our findings. First, our sample may not be representative of people who have been victims of child maltreatment as it was drawn from people participating in therapy in a large metropolitan area. Prior research suggests there are gendered differences in the internalization and externalization of behaviors related to child abuse and neglect (Jung et al., 2015; Tyler et al., 2008), but our data do not allow us to explore these differences. The majority of our participants identified as female, thus male participants may be underrepresented. Although our sample reflects the White and Hispanic demographics of the area, it is not representative of other groups such as Black, Asian/Pacific Islander, or Middle Eastern populations. Research suggests there are important cultural differences in acceptable child-rearing behaviors and reporting practices (Cerdeña et al., 2021; Chan et al., 2011; Ibanez et al., 2006).

Second, respondents were recruited from people participating in a trauma therapy and support group. As described in the methodology, the majority of participants had reported experiencing childhood sexual abuse. As these experiences are not broadly encompassing of the myriad forms of childhood maltreatment, this may have skewed the impacts on participants’ social bonds, protective factors, and resilience. Finally, although the respondents were more likely to feel comfortable disclosing details about their various experiences with child maltreatment, there is an inherent socioeconomic bias. This is due in part to the population from which are sample was drawn, i.e., people participating in therapy. Thus, it is possible that people who cannot afford therapy may have different experiences. The authors also acknowledge the limitations of research based on interview data in general including social desirability bias and the subjective nature of the data and data analysis which may limit validity, reliability, and generalizability of the findings. Given the bias of the recruitment process and the small sample size of the study, the authors make no claims as to the generalizability of our findings to all child maltreatment survivors. However, the data do provide insight into childhood maltreatment victimization and survivors for the perspective of survivors, and the authors have no reason to doubt their experiences as they have described them.

### Policy Implications

The results of the data analyses suggest the application of early intervention and prevention programs in the effort to reduce and inhibit antisocial behaviors, and encourage prosocial, resilient behaviors (Anda et al., 2006; Ban & Oh, 2016; Butler et al., 2020; Benedini & Fagan, 2018; CDC, 2024; Cho et al., 2024; Craig et al., 2025; Dube et al., 2003; Felitti et al., 1998; Lieberman et al., 2011; Mielke et al., 2020; Özdemir Bişkin, 2024; Roettger & Dennison, 2018; Su et al., 2022; Ungar, 2013; Zettler, 2020). Possible policy implementations could include programs and campaigns that aim to bring awareness within communities about the effects of childhood maltreatment. Many parents may be unaware of the expansive kinds of childhood maltreatment, as well as their short-term and long-term impacts (CDC, 2024; Dube et al., 2003). Such opportunities allow for the rebuilding of attachment bonds between parents and their children, as well (Lowell et al., 2014; Widom et al., 2018; Wright & Folger, 2017).

Sliding-scale community health clinics can promote mental and physical health and wellness (Anda et al., 2006; Bürgin et al., 2025; Kramer et al., 2008; Zettler, 2020). Pediatric screenings in-tandem with school-based screenings for childhood maltreatment can flag at-risk children may also be implemented (Ban & Oh, 2016; Felitti et al., 1998; Su et al., 2022). Parents can be provided with resources such as parenting guides, classes, support groups, and counseling (Kramer et al., 2008; Lowell et al., 2014; Özdemir Bişkin, 2024; Su et al., 2022; Zettler, 2020). By offering accessible screenings within communities, parents may have be provided with proper resources to alleviate their own childhood trauma and associated stressors, as well as helping their children (Lowell et al., 2014; Mielke et al., 2020; Widom, 1989). School- and community-based treatments such as multisystemic therapy and cognitive behavioral therapy can incorporate multiple levels of protective factors, in the effort to provide multiple easily accessible support systems (Afifi & MacMillan, 2011; Baldwin et al., 2023; Ban & Oh, 2016; Bürgin et al., 2025; Cerdeña et al., 2021; Cho et al., 2024; Cui et al., 2020; Mc Gee et al., 2020; Meng et al., 2018; Ungar, 2013; Zettler, 2020).

### Future Research

Future research should continue to focus on why certain individuals refrain from antisocial behavior, despite the childhood maltreatment they experienced (Benedini & Fagan, 2018; Zhang et al., 2026). Studies exploring the role of social bonds with large, representative samples should take into consideration the gendered and cultural differences in childhood maltreatment. This would contribute to our understanding of the nature of childhood maltreatment, and how to best address it through policy (Cerdeña et al., 2021; Chan et al., 2011; Ibanez et al., 2006; Jung et al., 2015; Tyler et al., 2008; Zhang et al., 2026). The findings suggest that involvement is crucial in early childhood and adolescence for helping victims of child abuse and neglect form social bonds that reduce the likelihood of participation in antisocial behavior and for facilitating resiliency. This is an important avenue for future research to explore.

## Conclusion

This study highlighted the relationships between childhood maltreatment and protective factors of social bonds and resilience. The current study aimed to demonstrate how some survivors refrain from offending and perpetuating the cycle of violence onto others, and how various protective factors mitigate the long-term impacts of childhood maltreatment (Butler et al., 2020; Widom et al., 2018; Zhang et al., 2026). Although there were pertinent limitations of the study, the current study aimed to link and illustrate the intersectionality of childhood maltreatment, protective factors, social bonds, and resilience. Furthermore, the data analyses and findings of the study provide suggestions for future research opportunities, which can hopefully shed further light on promoting resilience and breaking the cycle of violence for future generations.

## Appendix

### Interview Guide

1. Please describe your past child abuse and/or neglect.
  - Make more nuanced and “ease into it”: Why did you contact Therapist/start going to therapy?
  - When did it happen? How did it happen? How many times?
    i. Who, what, where, when, why, and how. (might be multiple abusers)
  2. Describe how you believe that your past child abuse and/or neglect affected your quality of life during childhood and adolescence. Ask for very specific examples. Tell a story. Can you remember a specific example? For example, where you got upset at school or a party or family gathering because of the abuse?
2. Describe how you believe that your past child abuse and/or neglect affected your interpersonal relationships with others. Specific example follow-up questions. Family, friend, boyfriend? How did they affect your relationships?
3. Have you ever abused/victimized someone? Has your abuse ever caused you to abuse/violate other people? Refer to the cycle of violence.
4. Describe how you believe that your past child abuse and/or neglect affected your criminal or deviant behavior during adolescence, if any. Did your abuse/neglect cause you to engage in crime? If so, how? Give me an example. Can you explain?
5. Describe how you believe that your past child abuse and/or neglect affected your quality of life during adulthood so far. Give specific examples and details. Job? Home? Relationships?
6. Describe your personal reasons for why you refrain criminal behavior during adulthood. Link into resilience.
7. Describe what past factors have positively impacted your life and influenced your behavior during your adolescence/adulthood.
8. Describe what past experiences or relationships from your childhood or adolescence have positively impacted your life and influenced your behavior during your adulthood. Ask for details.
9. Describe your personal attributes or characteristics that you believe positively impacted your life and influenced your behavior during your adulthood.
10. Do you believe you are still struggling with any of the long-term effects of the abuse and neglect you experienced during your childhood?
11. Describe why you believe your personal characteristics and/or these protective social factors may have influenced your adult behavior and quality of life.
12. Explain the resiliency concept to them, then ask them to explain to you if they agree or disagree with it and why and to give you specific examples from their experiences. General influence on your adulthood.
13. Demographic Questions:
  - Age, ethnicity/race, gender, education attainment, last year of school they finished, marital status, employment status, religious affiliation.
  - Criminal history: Have you ever been arrested? If so for what? Have you ever been convicted of a crime? If so for what? Specific examples? Have you committed any non-traffic crimes that you weren’t arrested for? If so, what? How many times have you committed that crime in the last year? Two years? Three years?

## Footnotes

1 Approval for the study was obtained from the University’s Institutional Review Board before the recruiting process (Ethics approval number: IRB-23-594). As participation in this study was voluntary, respondents were not provided compensation.
